# PERI-operative biologic DMARD management: Stoppage or COntinuation during orthoPaEdic operations (the PERISCOPE trial) – a study protocol for a pragmatic, UK multicentre, superiority randomised controlled trial with an internal pilot, economic evaluation and nested qualitative study

**DOI:** 10.1136/bmjopen-2024-084997

**Published:** 2024-06-22

**Authors:** Samantha Brady, Andrew Mott, Katie Carlisle, Abhishek Abhishek, Joy Adamson, Laura Coates, Bernard van Duren, Paul Emery, Susan Marion Goodman, Catherine Hewitt, Jinshuo Li, Laura Mandefield, Gillian Parkinson, Helena Marzo-Ortega, James Maxwell, Jagdeep Nanchahal, Amar Rangan, Duncan Richards, Sarah Ronaldson, Susan Shepherd, Johanna Taylor, Jeremy Mark Wilkinson, Hemant Pandit, Kulveer Singh Mankia

**Affiliations:** 1 Department of Health Sciences, University of York, York, UK; 2 Academic Rheumatology, University of Nottingham, Nottingham, UK; 3 NDORMS, Oxford Brookes University Faculty of Health and Life Sciences, Oxford, UK; 4 Leeds Institute of Rheumatic and Musculoskeletal Medicine, University of Leeds, Leeds, UK; 5 Leeds NIHR Biomedical Research Centre, Leeds Teaching Hospitals NHS Trust, Leeds, UK; 6 Cornell University Joan and Sanford I Weill Medical College, New York City, New York, USA; 7 Sheffield Teaching Hospitals NHS Foundation Trust, Sheffield, UK; 8 Kennedy Institute, The University of Oxford, Oxford, UK; 9 University of Oxford, Oxford, UK; 10 NDORMS, University of Oxford, Oxford, UK; 11 School of Medicine and Population Health, University of Sheffield, Sheffield, UK; 12 Chapel Allerton Hospital, Leeds, UK

**Keywords:** orthopaedic & trauma surgery, rheumatology, randomized controlled trial

## Abstract

**Introduction:**

Biological disease-modifying antirheumatic drugs (bDMARDs) have revolutionised the treatment of inflammatory arthritis (IA). However, many people with IA still require planned orthopaedic surgery to reduce pain and improve function. Currently, bDMARDs are withheld during the perioperative period due to potential infection risk. However, this predisposes patients to IA flares and loss of disease control. The question of whether to stop or continue bDMARDs in the perioperative period has not been adequately addressed in a randomised controlled trial (RCT).

**Methods and analysis:**

PERISCOPE is a multicentre, superiority, pragmatic RCT investigating the stoppage or continuation of bDMARDs. Participants will be assigned 1:1 to either stop or continue their bDMARDs during the perioperative period. We aim to recruit 394 adult participants with IA. Potential participants will be identified in secondary care hospitals in the UK, screened by a delegated clinician. If eligible and consenting, baseline data will be collected and randomisation completed. The primary outcome will be the self-reported PROMIS-29 (Patient Reported Outcome Measurement Information System) over the first 12 weeks postsurgery. Secondary outcome measures are as follows: PROMIS - Health Assessment Questionnaire (PROMIS-HAQ), EQ-5D-5L, Disease activity: generic global Numeric Rating Scale (patient and clinician), Self-Administered Patient Satisfaction scale, Health care resource use and costs, Medication use, Surgical site infection, delayed wound healing, Adverse events (including systemic infections) and disease-specific outcomes (according to IA diagnosis). The costs associated with stopping and continuing bDMARDs will be assessed. A qualitative study will explore the patients’ and clinicians’ acceptability and experience of continuation/stoppage of bDMARDs in the perioperative period and the impact postoperatively.

**Ethics and dissemination:**

Ethical approval for this study was received from the West of Scotland Research Ethics Committee on 25 April 2023 (REC Ref: 23/WS/0049). The findings from PERISCOPE will be submitted to peer-reviewed journals and feed directly into practice guidelines for the use of bDMARDs in the perioperative period.

**Trial registration number:**

ISRCTN17691638.

**STRENGTHS AND LIMITATIONS OF THIS STUDY**

- Broad eligibility criteria will allow us to generalise our findings to patients with different types of inflammatory arthritis undergoing different orthopaedic surgical procedures.
- Embedded qualitative work will allow us to explore patient and clinician views on the interventions and perceptions of risk.
- The primary outcome covers multiple aspects of health-related quality of life to see how the interventions affect patients’ lives over the first 12 weeks following surgery allowing us to assess differences in recovery.
- A range of secondary outcomes will assess disease severity, pain, infections, wound healing, adverse events and costs.
- This study excludes some patients with a history of infections or who are taking additional medications that might impact infection risk, as these may impact some of the outcomes collected.

## Introduction

 Inflammatory arthritis (IA) affects around 1% of the population and includes rheumatoid arthritis (RA), psoriatic arthritis (PsA), axial spondyloarthritis (axSpA) and juvenile IA (JIA).^1 2^ Over 400 000 people in the UK have RA, and in North America, 7 million people are affected, often with a significant impact on quality of life.^3^ A significant proportion of people with IA require long-term biological disease-modifying antirheumatic drugs (bDMARDs) that reduce inflammation by targeting the immune response. Although these drugs limit disease severity and progression, many patients continue to require planned orthopaedic surgical intervention to manage pain and restricted function caused by joint and tendon damage.^4^,^7^ It remains unclear whether patients with IA undergoing surgery are at an increased risk of surgical site infection (SSI) and/or delayed wound healing.^6^,^8^ The severe consequences associated with infection following orthopaedic surgery led the British Society of Rheumatology (BSR), American College of Rheumatology (ACR) and American Association of Hip and Knee Surgeons to recommend withholding bDMARDs during the perioperative period, despite a lack of definitive evidence.^9^,^11^

Withholding bDMARD treatment puts patients at an increased risk of disease flares in the perioperative period, but the magnitude of the risk is poorly characterised.^12^ Disease flares delay recovery, impact the overall quality of life and can severely compromise overall disease control.^13^ Avoiding disease flares in the perioperative period aids timely rehabilitation. If flares occur and disease control is lost, patients are often managed with courses of corticosteroids. Although effective in treating flares, corticosteroids increase the risk of infection in a dose-dependent fashion.^14 15^ Therefore, using the lowest possible corticosteroid dosage to ensure stable IA during the perioperative period is recommended.^16^ How best to balance the relative risks of perioperative infection and disease flare remains to be established and, therefore, the question of whether to stop or continue bDMARDs in the perioperative period has not been adequately addressed.

Based on the available published data, Goodman *et al*^11^ published the ACR guidelines for perioperative management of antirheumatic medication in patients with IA. They recommended that in cases of RA, AxSPa, PsA and JIA, clinicians should continue the current dose of non-biological DMARDs (such as methotrexate) for patients undergoing elective hip or knee replacement (arthroplasty). They noted that the randomised controlled trials (RCTs) comparing continuation versus stoppage of DMARDs in the perioperative period revealed that the risk of infection was decreased, not increased, when DMARDs were continued, with RR of 0.39 (95% CI 0.17 to 0.91).

The recent increased availability and the use of biologics in IA has brought the perioperative management of bDMARDs into sharper focus.^17^ These patients need optimal disease control to reduce the risk of flares and enable active engagement in postoperative rehabilitation, a key requisite to achieve timely recovery and optimal restoration of function. However, there is a lack of robust data regarding the safety of continuation of bDMARDs in the perioperative period.

### Objectives

1. Assess whether continuation of bDMARDs is superior to stoppage with respect to postoperative health-related quality of life (HRQoL).
2. Investigate the difference between bDMARDs stoppage versus continuation for a range of secondary outcomes, including physical function, HRQoL, disease activity, medication use, healthcare resource use and surgical outcomes (see below).
3. Conduct a cost-effectiveness analysis.
4. Undertake a qualitative study involving patients and clinical staff.

### Trial design

The PERISCOPE trial is a UK multicentre, superiority RCT with an internal pilot, economic evaluation and nested qualitative study, investigating the stoppage or continuation of bDMARDs in the perioperative period.

## Methods

### Participants, interventions and outcomes

**Figure 1:**
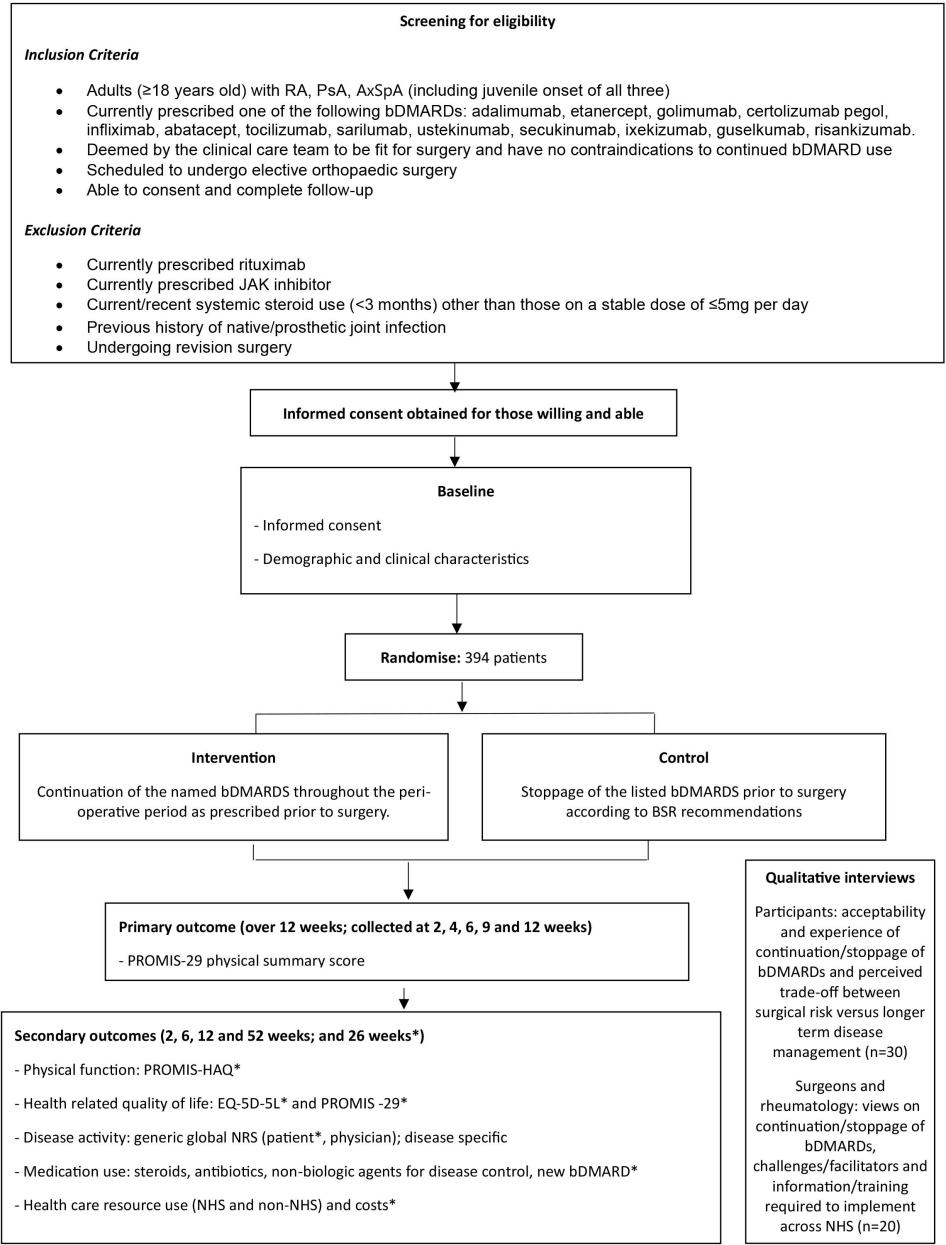
Participant timeline. *Outcomes which occur at the 26 week visit. axSpA, axial spondyloarthritis; bDMARDs, biological disease-modifying antirheumatic drugs; BSR, British Society of Rheumatology; JAK, Janus Kinase; NHS, National Health Service; PROMIS-HAQ, Patient Reported Outcome Measurement Information System - Health Assessment Questionnaire; PsA, psoriatic arthritis; RA, rheumatoid arthritis.

The PERISCOPE trial will be set within National Health Service (NHS) secondary care in approximately 20 hospitals in the UK. The planned recruitment period is April 2022–April 2025. Figure 1 shows the participant flow through the trial.

### Eligibility criteria

#### Inclusion criteria

- Adults aged 18 years and over.
- Diagnosed with RA, PsA or axSpA (including juvenile onset of all three).
- Currently prescribed one of the following bDMARDs: TNF inhibitors (adalimumab/etanercept/golimumab/certolizumab pegol/infliximab); CTLA4-Ig (abatacept); IL-6 inhibitors (tocilizumab/sarilumab); IL-12/23 inhibitors (ustekinumab); IL-17 inhibitors (secukinumab/ixekizumab); IL-23 p19 inhibitors (guselkumab/risankizumab).
- Considered by the clinical care team to be fit for surgery and have no contraindications to continued bDMARD use.
- Scheduled to undergo elective orthopaedic surgery (soft tissue surgery, joint replacement or other metalwork implantation).
- Able to consent and complete follow-up.

#### Exclusion criteria

- Currently prescribed JAK inhibitors.
- Currently prescribed rituximab.
- Prescribed systemic steroids (within 3 months of planned surgery date) other than those on a stable dose of ≤5 mg per day.
- The use of intramuscular or intra-articular injections will remain at the clinician discretion.
- Previous history of native/prosthetic joint infection.
- Undergoing revision surgery.
- Current pregnancy.

### Obtaining informed consent

Potentially eligible participants will be identified by screening waiting lists for orthopaedic surgery across the participating sites. Patients will be identified in combined rheumatology-orthopaedic multidisciplinary team clinics by a member of their direct clinical care team. In addition, patients with IA on bDMARDs presenting to secondary care with an orthopaedic problem requiring surgical intervention will be screened for eligibility by the local research teams and approached to establish if they are interested in participating in the study.

Potential participants will be provided with an invitation letter and a detailed participant information sheet, which will explain the risks and benefits of trial participation clearly, and a trial infographic. These may be given out in the clinic, emailed or sent by post. Sites will contact the participants by telephone to check their willingness to participate in the study and to answer any questions they may have. If they are interested in participating and are deemed eligible, they will meet with their clinician or research nurse who will fully discuss all points presented in the patient information sheet.

For patients willing and able to provide informed consent, consent will be recorded at the screening visit via paper consent forms, which will be uploaded onto the secure web-based data collection interface ‘REDCap’ once complete or via participant e-consent directly within the REDCap system. Informed consent will be obtained by a suitably qualified and experienced local research nurse or clinician who has been authorised to do so by the chief or principal investigator, as detailed on the study delegation log.

Study participation is voluntary, and participants can withdraw consent at any time without their legal or medical rights being affected.

### Intervention and usual care

#### Intervention

Participants will continue taking their bDMARDs throughout the perioperative period as prescribed prior to elective orthopaedic surgery. All other aspects of care will continue as per usual practice, including concomitant non-bDMARDs and postsurgical rehabilitation.

#### Usual care

Stoppage of bDMARDs prior to surgery and recommencing treatment after wound healing and removal of sutures/clips, according to BSR recommendations.

Given the pragmatic nature of the PERISCOPE trial, where a participant’s surgery is delayed (for medical or non-medical reasons), it will be at the discretion of the clinician as to whether bDMARDs should be recommenced while surgery is rescheduled as per current clinical practice.

Treatment can be restarted when there is evidence of good wound healing (normally after 2 weeks), all sutures and staples have been removed, and there is no evidence of infection.

Any modifications or changes to allocated interventions will be recorded and reported in the trial case report forms.

Following completion of their follow-up, participants will remain in the care of the treating clinicians as per usual clinical practice.

### Outcomes

#### Primary outcome

The primary outcome is Patient Reported Outcome Measurement Information System (PROMIS)-29 over the first 12 weeks postsurgery (2, 4, 6, 9, 12 weeks). The PROMIS-29 is a measure that encompasses seven domains including physical function, anxiety, depression, fatigue, sleep disturbance, ability to participate in social life and activities, and pain interference.^18^,^20^

#### Secondary outcomes

Secondary outcomes will all be collected at 2, 6, 12 and 52 weeks. Additionally, those outcomes marked with an asterisk will also be collected at 26 weeks. These include:

1. Physical function: PROMIS - Health Assessment Questionnaire (PROMIS-HAQ).*
2. HRQoL: EQ-5D-5L.*
3. Disease activity: generic global Numeric Rating Scale (NRS) (patient).*
4. Surgery/outcome satisfaction: Self-Administered Patient Satisfaction scale.*^21^
5. Healthcare resource use (NHS and non-NHS) and costs.*
6. Medication use (steroids, antibiotics, non-biological agents for disease control, change to or addition of a new DMARD).*
7. Disease activity: generic global NRS (physician).
8. SSI: modified 1992 Centers for Disease Control and Prevention criteria for postoperative infection.^22^
9. Delayed wound healing: A surgical wound will be considered as ‘healed’ if by 2 weeks postsurgery the surgical incision has healed by primary intention without any evidence of gaping or dehiscence. Any wound that has not healed fully by primary intention by 2 weeks postsurgery, will be considered as ‘delayed wound healing’.
10. Adverse events (AEs) including systemic infections.

*items collected additionally to the PROMIS-29 at the 26 week visit.

#### Disease-specific outcomes

Disease -specific outcomes will be collected at 2, 6, 12 and 52 weeks, which will include:

##### Rheumatoid arthritis

Clinical Disease Activity Index.

##### Axial spondylarthritis

1. Spinal pain: NRS.
2. Global Disease Activity score: NRS.
3. The Bath Ankylosing Spondylitis Disease Activity Index.
4. The Bath Ankylosing Spondylitis Functional Index.
5. Assessment of SpondyloArthritis International Society Health Index.

##### Psoriatic arthritis

1. 66/68 joint count measure.
2. Body surface area for skin.
3. Leeds Enthesitis Index.
4. Total dactylitis count.
5. Disease interference: NRS.

### Participant timeline

#### Sample size

The minimum clinically important difference (MCID) for the PROMIS-29 is not well established, however, the HAQ-DI has a well-established MCID in IA between 0.25 and 0.35 (SD=0.68).^23^ In a population with RA, when using patient anchors, the absolute change in PROMIS scores was generally 1–3 points, with a meaningful change associated with a change of 3–5 points.^20^ Within this population, the effect size of 0.37 (based on the HAQ-DI MCID) translates to an MCID of 3.7, which is in line with the early evidence. Assuming the same magnitude of effect for the PROMIS-29, with 90% power, 5% alpha, an effect size of 0.37 and 20% attrition, the target sample size is 394 randomised participants.

#### Recruitment

Strategies for achieving adequate participant recruitment will include seeking advice from our patient advisory group (PAG) and completing recruitment evaluation interviews with site teams.

Trial training and discussions in relation to key study elements will be implemented through online site investigator meetings.

Research teams will be provided with training at a site initiation visit. A trial manual will be provided to ensure adherence to trial processes. Support and guidance will be provided to staff when required (eg, when new staff join) with clinical guidance from the co-chief investigators when necessary.

### Assignment of interventions: allocation

#### Sequence generation

The allocation sequence will be generated using Stata (V.17). The sequence will allow for independent randomisation 1:1 (intervention:control), using block randomisation, stratified by underlying disease, type of surgery and sex.

The allocation sequence will be implemented using the REDCap system at the baseline visit.

The allocation schedule will be generated by a statistician at York Trials Unit (YTU) not involved in the recruitment of participants. Enrolment into the study will be completed by a suitably qualified local research nurse or clinician. Randomisation will be undertaken by local site staff using REDCap at the baseline visit, which will be scheduled to allow for at least one bDMARD dosing interval before the date of surgery.

Neither the PERISCOPE study design nor the interventions allow for the blinding of clinicians or participants, therefore, an unblinding procedure is not required for this study.

#### Data collection and management

##### Plans for assessment and collection of outcomes

Data collection will occur at 2, 4, 6, 9, 12, 26 and 52 weeks postsurgery. This will be completed either via REDCap or on paper questionnaires. Collection of data at 4, 9 and 26 weeks will be completed remotely, all others will be completed at clinic visits. The schedule of outcomes and time points is provided in online supplemental material 1.

At baseline, participants will complete the PROMIS-29, PROMIS-HAQ, EQ-5D-5L and an NRS of disease activity. Data will also be collected regarding participant demographics, disease and medication history, comorbidities, and disease-specific measures.

Data relating to the participant’s surgery will be collected once the surgery has been completed.

To facilitate retention participants will have the option of paper or electronic follow-up. Participants will receive a maximum of three reminders if follow-up is not returned to encourage completion.

Participants whose surgery is cancelled will be followed up from the date of cancellation, if surgery is delayed, follow-up will commence from the completion date of surgery.

### Data management

All data will be collected in REDCap using case report forms. Data will be held securely on the cloud-hosted REDCap server. Appropriate range checks and validation will be included to promote data quality. Access to the study interface will be restricted to named authorised individuals.

Confidentiality will be maintained by using unique identifiers for each participant.

The storage of personal data will be almost exclusively in electronic format. Where this may need to be in paper form (eg, completed consent forms for the qualitative study interviews, where consent forms are sent to patients electronically or in the post before verbal consent is confirmed over the phone), documentation will be kept in a locked filing cabinet or on a password protected computer in a locked office in compliance with the Data Protection Act (2018). This will be in a secure area that is only accessible to authorised research staff using a security pass at YTU, University of York.

### Statistical methods

#### Statistical methods for primary and secondary outcomes

Analyses will be conducted once at the end of the trial using the latest available version of Stata/SE. No formal interim analyses will be undertaken during the trial. At the end of the internal pilot (9 months), the trial progress will be assessed against predetermined criteria (detailed in online supplemental material 2) to assess the suitability of continuing to a full trial or an early termination.

The primary analysis will be by intention to treat and will follow Consolidated Standards of Reporting Trials (CONSORT) reporting guidelines for a superiority study. A detailed statistical analysis plan (SAP) will be written before the follow-up period concludes.

Continuous variables will be summarised in terms of the available sample size (the number of individuals with non-missing data), arithmetic mean, SD, median, IQR, minimum and maximum. Categorical data will be summarised at the individual level in terms of frequencies and proportions.

PROMIS scores will be calculated using the Health Measures Scoring Service.^24^ This method of scoring uses responses to each item for each participant and is useful when there is missing data. It is preferred to the alternative manual scoring as it is more accurate than the use of raw score/scale score look-up tables.

In the primary analysis, we will compare the primary outcome between groups using a covariance pattern mixed-effect linear regression model, incorporating postrandomisation time points. Treatment groups, time point, treatment-by-time interaction and baseline covariates will be included as fixed effects. Participants will be included as a random effect accounting for repeated observations per patient. Point estimates and 95% CIs will be extracted from the model with the estimate over 12 weeks as the primary outcome of interest. In the primary analysis model, any missing outcome data will be assumed to be missing at random.

Continuous secondary outcome measures will be analysed using the same type of model as that for the primary outcome. Delayed wound healing and SSIs will be compared using a generalised linear model; risk differences and relative risk will be reported. Harms will be reported descriptively, including the number and nature of (serious) AEs and number of participants with at least one (serious) AE. Medication use will also be reported descriptively.

For the statistical analyses described above, participants will be analysed as part of the groups to which they were randomised, regardless of subsequent adherence to the allocated condition. A summary of any protocol deviations will be provided.

Participant-level datasets and statistical code used during the analysis of the study may be shared on reasonable request following the publication of the main results paper.

### Methods for additional analyses (eg, subgroup analyses)

Two exploratory subgroup analyses will be undertaken to investigate the potential differential effect of the intervention by the type of surgery and underlying disease by adding an interaction term between the relevant factor and randomised group in the primary analysis model.

For the primary outcome measure, patterns of missingness will be explored and a sensitivity analysis will be considered to assess any departures from the missing at random assumption.

#### Health economic analysis

The cost-effectiveness of bDMARD continuation compared with bDMARD stoppage will be evaluated using a within-trial cost–utility analysis, from the perspective of the NHS and personal social services, over 12 months. The initial surgery will be costed using matching HRG4+codes.^25^ Data on EQ-5D-5L, primary and secondary healthcare resource use will be collected over a 12-month period, using self-completed questionnaires (at baseline, 2, 6, 12, 26 and 52 weeks postrandomisation) and hospital case report forms. Costs regarding medication use will also be incorporated (ie, antibiotics, bDMARDs and other medications for disease control). Unit costs will be obtained from established costing sources of the appropriate year^26^,^28^ and attached to each resource item/medication to generate total cost estimates for each participant. Further data will be collected for a secondary analysis, to explore the impact on private expenditures (ie, out-of-pocket medication expenditure, travel costs for appointments) and lost productivity on cost-effectiveness findings.

Health outcomes will be measured in terms of quality-adjusted life-years (QALYs), derived from the EQ-5D-5L^29^ in the base case. The total QALYs accrued by each participant during the 12 months will be estimated using the area under the curve method.^29^ Missing data patterns will be examined and used to guide the multiple imputation methods employed to deal with missing data.^30^ Incremental costs and QALYs will be estimated by means of regression methods, allowing for correlation between costs and utilities, and adjusting for key covariates. An incremental cost-effectiveness ratio (ICER) will be calculated if both incremental costs and QALYs are positive and net health benefit will be calculated over the maximum acceptable ICER threshold range at £20 000—£30 000 per QALY gained.^31^ Non-parametric bootstrapping will be used to explore uncertainties surrounding mean estimates, accounting for skewed data. CIs and cost-effectiveness acceptability curves^32^ will be constructed using bootstrapped estimates to describe uncertainty around the analysis findings. Sensitivity analyses will explore the impact of using PROMIS-29^33^ to derive QALYs, varying key cost parameters and assumptions underpinning the analysis model on the conclusions. Analyses will take an intention-to-treat approach. If deemed appropriate (ie, dependent on the trial results and data availability), the economic findings will be extrapolated beyond the trial’s 12-month time horizon.

#### Nested qualitative study

The aim of the nested qualitative study is to explore the patients’ and clinicians’ acceptability and experience of continuation/stoppage of bDMARDs in the perioperative period and the impact postoperatively.

#### Qualitative study sample

A purposive sample of up to 30 participants (approximately 25 trial participants and 5 who declined to participate in the trial) will be included in the qualitative study. The participants will be sampled on sociodemographic characteristics, underlying disease and randomised group to ensure maximum variation.

Semistructured interviews will also be conducted with up to 10 orthopaedic surgeons, up to 10 rheumatologists and up to 10 clinicians at sites who decline to participate.

#### Consent to qualitative study

Patients who consent to the main trial will be provided with an optional consent statement on the consent form to consent to the qualitative study. Patients approached for consent who do not wish to participate in the trial will be approached to participate in the qualitative study and complete a separate consent form.

For all participants taking part in the qualitative study, they will be reminded at the start of the interview, prior to recording, what the interview entails, given an opportunity to ask questions and reminded of their right to withdraw at any time. Once the audio recording has started, they will be asked to confirm they are willing to continue.

Clinicians who are taking part and those who decline to participate in the trial will also be approached for an interview by the research team. They will be provided with an information sheet and the opportunity to ask any questions. Clinicians that agree to participate will be emailed a consent form. Prior to the commencement of a telephone or video interview, the researcher will ask for the participant’s verbal consent to each item on the written consent form. Taking of this verbal consent will be audio recorded.

#### Qualitative study data collection

Semistructured interviews will be conducted with the patients at around 3 to 6 months postsurgery. Interviews undertaken with clinicians will be undertaken at various times throughout recruitment. These will be audio recorded using an encrypted device.

Recordings of initial meetings held between the trial management team and interested recruiting sites will be collected. From these, a purposive sample of declining site investigators will also be interviewed.

#### Qualitative data analysis

Qualitative data analysis will follow the principles of thematic analysis, providing an interpretive exploration of the experiences, attitudes and beliefs of different stakeholder groups.^34 35^ Emerging codes and themes will be discussed as a team and at regular intervals with the PAG.

#### Qualitative study data management

All interviews will be digitally audio recorded (with consent), anonymised and transcribed, the transcripts forming the data for analysis. Audio data will be removed from recording devices as soon as possible after transfer to secure, password-protected servers at YTU. The audio recordings, transcripts and other data capture forms will be kept securely as described with other documentation not held electronically on REDCap.

Interview data will be pseudoanonymised and stored for a minimum of 10 years.

### Patient and public involvement

The PERISCOPE protocol was developed with the PAG, including inclusion and exclusion criteria, study schedule and primary and secondary outcome measures, including outcome assessment tools, as well as ways to support diversity and inclusivity in PERISCOPE.

The PAG will work with the study team to enhance recruitment by codeveloping study documents and communication tools (written and pictorial). They will ensure dissemination of findings is accessible and engaging for patients, their carers and the public, including historically underserved communities.

The PAG will meet regularly throughout the study. Two patient and public involvement (PPI) member places will be reserved on each of the trial steering committee (TSC) and trial management group (TMG), with only one PPI member required to attend each meeting.

### Oversight and monitoring

#### Composition of the coordinating centre and TSC

YTU will oversee and coordinate the day-to-day management and running of the study. This will include trial management and coordination, statistical, economic and data management staff.

The TMG will meet bimonthly and will consist of the chief and coinvestigators, and members of YTU responsible for the study.

The TSC will comprise an independent chair (methodologist), two public/patient contributors, a consultant orthopaedic surgeon and a consultant rheumatologist who are also independent of the study research team. The TSC will provide overall supervision for the trial on behalf of the sponsor and funder.

#### Composition of the data monitoring committee, its role and reporting structure

The PERISCOPE data monitoring and ethics committee (DMEC) is independent of the study sponsor and comprises a statistician (chair) and independent clinicians (rheumatologist and orthopaedic surgeon). All members of the DMEC have signed a DMEC charter and confirmed they have no competing interests. This is stored in the trial master file at YTU.

The DMEC will meet every 6 months (or more frequently if the committee requests) to provide project oversight to the trial. This will include monitoring safety and efficacy data as well as quality and compliance data and ensuring that the protocol is accurately followed, and the study is GCP compliant. The committee will recommend whether there are any ethical or safety reasons why the trial should not continue and report these in writing to the TSC. The independent members of the DMEC committee will be allowed to see unblinded data.

#### AE reporting and harms

AEs associated with the trial treatment (intervention or control) will be recorded throughout the trial in case report forms irrespective of whether they are expected or unexpected. Possible AEs could include SSI, systemic infection and venous thromboembolism.

Serious AEs that are deemed related to the research and are unexpected will be reported to the Research Ethics Committee (REC). All AEs will be routinely reported to the TMG, TSC and DMEC. The DMEC will be responsible for reviewing related and unexpected serious AEs.

All AEs will be reported in relevant trial publications.

#### Frequency and plans for auditing trial conduct

Regular central monitoring will be performed according to ICH GCP (International Council for Harmonisation of Technical Requirements for Registration of Pharmaceuticals for Human Use Good Clinical Practice) and the PERISCOPE monitoring plan. The PERISCOPE monitoring plan which will be agreed by the sponsor, TMG, TSC and chief investigators. Data will be evaluated for compliance with the protocol and GCP and the applicable regulatory requirements.

### Plans for communicating important protocol amendments to relevant parties

Substantial protocol changes will first be agreed on the funding body, sponsor, TSC, DMEC and TMG. For minor changes to the protocol agreement will be sought from the TMG and sponsor. Amendments will then be made to the required documentation and the Health Regulatory Authority (HRA) amendment tool completed. This tool will confirm the category of the amendment. Once sponsor authorisation has been confirmed, YTU will submit via IRAS and, where necessary, obtain approval from the REC, HRA and host institution(s) for approval of all substantial amendments to the original approved documents. Once approvals are received, the new documents/versions will be shared with sites and the study version control log will be updated for sites to check they are using only the most recent versions of trial documents.

For any amendments to trial eligibility criteria, the ISRCTN registry will also be updated.

Trial participants will be written to, if necessary, to explain any changes.

### Ethics and dissemination plan

Ethical approval for this study was sought and received from the West of Scotland REC on 25 April 2023 (REC Ref: 23/WS/0049).

The findings from PERISCOPE will be submitted to peer-reviewed journals and will feed directly into practice guidelines for the use of biologics at the time of orthopaedic surgery. Dissemination will focus on supporting the wider adoption and implementation of the research findings. The trial results, alongside findings from the qualitative work, will inform the optimal approach to how the evidence should be described to key stakeholders, in order to facilitate patient and clinician decision-making as part of high-quality patient-centred care.

All key protocol contributors will be provided the opportunity to fulfil International Committee of Medical Journal Editors author criteria. Details of planned publications and requirements for authorship will be detailed in a publication policy.

### Trial status

Recruitment to the PERISCOPE Trial began in June 2023 and will be complete by 30 April 2025.

## supplementary material

10.1136/bmjopen-2024-084997 online supplemental file 1

10.1136/bmjopen-2024-084997 online supplemental file 2
