# Supplementary material for: PERI-operative biologic DMARD management: Stoppage or COntinuation during orthoPaEdic operations (the PERISCOPE trial) – a study protocol for a pragmatic, UK multicentre, superiority randomised controlled trial with an internal pilot, economic evaluation and nested qualitative study
Source: BMJ Open. 2024 Jun 22;14(6):e084997. doi: 10.1136/bmjopen-2024-084997 (PMC11328622; doi:10.1136/bmjopen-2024-084997)
Supplement: online supplemental file 1 [file bmjopen-14-6-s001.pdf]

Supplementary material 1: Schedule of outcomes and timepoints

TIMEPOINTS									
	Screening	Baseline	2 weeks	4 weeks	6 weeks	9 weeks	12 weeks	26 weeks	52 weeks
**Participant assessments**									
Demographic information		X							
PROMIS-29		X	X	X	X	X	X	X	X
PROMIS-HAQ		X	X		X		X	X	X
EQ-5D-5L		X	X		X		X	X	X
Surgery/outcome Patient satisfaction scale			X		X		X	X	X
Disease activity using a general global NRS (patient)		X	X		X		X	X	X
Health Resource use		X	X		X		X	X	X
Disease Specific Outcome Measures *		X	X		X		X		X
**Investigator assessments**									

Eligibility Confirmation	X								
Medication use		X	X		X		X		X
Disease activity using a generic global NRS (physician)		X	X		X		X		X
Disease specific outcome measures *		X	X		X		X		X
A modified 1992 Centre for Disease Control and prevention criteria for postoperative infection			X		X		X		X
Assessment for delayed wound healing			X		X		X		X
Adverse events			X		X		X		X

* Disease specific outcomes: **RA**: CDAI; **AS**: BASFI, BASDAI, ASAS-HI, NRS; **PsA:** 66/68 joint count, BSA for Skin, LEI, NRS
