# Supplementary material for: PERI-operative biologic DMARD management: Stoppage or COntinuation during orthoPaEdic operations (the PERISCOPE trial) – a study protocol for a pragmatic, UK multicentre, superiority randomised controlled trial with an internal pilot, economic evaluation and nested qualitative study
Source: BMJ Open. 2024 Jun 22;14(6):e084997. doi: 10.1136/bmjopen-2024-084997 (PMC11328622; doi:10.1136/bmjopen-2024-084997)
Supplement: online supplemental file 2 [file bmjopen-14-6-s002.pdf]

Supplementary material 2: Proposed progression criteria to be assessed at end of 9 month internal pilot

Progression criteria	Target at end of internal pilot	Green	Amber	Red
**Centres open**	8	100%<br>(8)	60-99 (5-7)	<60 (<5)
**Participant recruitment**	1-2 patients per site per month<br><br>79 Participants recruited	100%<br>(1-2)<br><br>100%<br>(79)	60-99<br>(0.6-<1-2)<br><br>60-99<br>(47-78)	<60 (<0.6)<br><br><60<br>(<47)
**Primary outcome data available**	At least 80%*	100%<br>(32)	80-99<br>(26-<32)	<80 (<26)

*Including participants recruited in the first 6 months
