# Supplementary material for: PERI-operative biologic DMARD management: Stoppage or COntinuation during orthoPaEdic operations (the PERISCOPE trial) – a study protocol for a pragmatic, UK multicentre, superiority randomised controlled trial with an internal pilot, economic evaluation and nested qualitative study
Source: BMJ Open. 2024 Jun 22;14(6):e084997. doi: 10.1136/bmjopen-2024-084997 (PMC11328622; doi:10.1136/bmjopen-2024-084997)
Supplement: Supplementary file 3 [file bmjopen-2024-084997.reviewer_comments.pdf]

PEER REVIEW HISTORY

BMJ Open publishes all reviews undertaken for accepted manuscripts. Reviewers are asked to complete a checklist review form (**http://bmjopen.bmj.com/site/about/resources/checklist.pdf**) and are provided with free text boxes to elaborate on their assessment. These free text comments are reproduced below.

ARTICLE DETAILS

**TITLE (PROVISIONAL)**	PERI-operative biologic DMARD management: Stoppage or COntinuation during orthoPaEdic operations: The PERISCOPE trial. A study protocol for a pragmatic, UK multi-centre, superiority randomised controlled trial with an internal pilot, economic evaluation and nested qualitative study
**AUTHORS**	Brady, Samantha; Mott, Andrew; Carlisle, Katie; Abhishek, Abhishek; Adamson, Joy; Coates, Laura; van Duren, Bernard; Emery, Paul; Goodman, Susan; Hewitt, Catherine; Li, Jinshuo; Mandefield, Laura; Parkinson, Gillian; Marzo-Ortega, Helena; Maxwell, James; Nanchahal, Jagdeep; Rangan, Amar; Richards, Duncan; Ronaldson, Sarah; Shepherd, Susan; Taylor, Johanna; Wilkinson, J. Mark; Pandit, Hemant; Mankia, Kulveer

This article was not externally reviewed at BMJ Open. Protocols that have been independently assessed prior to submission to BMJ Open are usually fast-tracked to publication on the grounds that further substantial changes will not be possible. This independent assessment will usually be external, independent review for both a substantial grant award from a non-commercial or government funder and ethics approval.
